# A case of neonatal sweet syndrome associated with mevalonate kinase deficiency

**DOI:** 10.1186/s12969-023-00887-8

**Published:** 2023-09-12

**Authors:** Margaret Irwin, Veeraya K. Tanawattanacharoen, Amy Turner, Mary Beth F. Son, Rebecca C. Hale, Craig D. Platt, Juan Putra, Birgitta A.R. Schmidt, Mollie G. Wasserman

**Affiliations:** 1 Department of Pediatrics, Boston Children’s Hospital, Harvard Medical School, 300 Longwood Ave, Boston, MA 02115 USA; 2 Division of Gastroenterology, Hepatology and Nutrition, Boston Children’s Hospital, Boston, MA USA; 3 Division of Rheumatology, Boston Children’s Hospital, Boston, MA USA; 4 Division of Immunology, Boston Children’s Hospital, Boston, MA USA; 5 Division of Hospital Medicine, Boston Children’s Hospital, Boston, MA USA; 6 Department of Pathology, Boston Children’s Hospital, Boston, MA USA

**Keywords:** Sweet syndrome, Mevalonate kinase deficiency, Mevalonate kinase-associated diseases, Very early-onset inflammatory bowel disease

## Abstract

**Background:**

Sweet syndrome (SS), also known as acute febrile neutrophilic dermatosis, is an immunologic syndrome characterized by widespread neutrophilic infiltration. Histiocytoid Sweet syndrome (H-SS) is a histopathologic variant of SS. While SS most commonly occurs in adults, this case report discusses an infant patient who presented with H-SS.

**Case presentation:**

Through a multidisciplinary approach, this patient was also found to have very early onset inflammatory bowel disease (VEO-IBD) and Mevalonate kinase-associated disease (MKAD). While prior case studies have characterized an association between VEO-IBD and MKAD, there is no literature describing the association of all three diagnoses this case: H-SS, VEO-IBD and MKAD. Initiation of canakinumab in this patient resulted in successful control of the disease.

**Conclusions:**

This case highlights the importance of a multidisciplinary approach to rare diagnoses, and collaboration during cases with significant diagnostic uncertainty.

**Supplementary Information:**

The online version contains supplementary material available at 10.1186/s12969-023-00887-8.

## Background

### Sweet Syndrome

Sweet syndrome, also known as acute febrile neutrophilic dermatosis, is an immunologic syndrome characterized by widespread neutrophilic infiltration. Sweet syndrome most commonly occurs in adults, often associated with either malignancy or drug exposure (most commonly granulocyte-colony stimulating factor) [1]. In addition, Sweet syndrome can be associated with respiratory and gastrointestinal conditions (i.e., upper respiratory or gastrointestinal tract infections) and pregnancy. It most commonly presents with fever followed by cutaneous manifestations, which begin as painful, erythematous papules and nodules that can progress to asymmetrically distributed plaques. The diagnosis is confirmed by histopathology, classically showing dense neutrophilic infiltration without an infectious etiology. Histopathologic variants have been described including histiocytoid Sweet syndrome, where the histologic infiltrate is dominated by histiocytoid mononuclear cells [2, 3]. Sweet syndrome is highly responsive to systemic corticosteroids but is also known to resolve spontaneously.

Neonatal Sweet syndrome is exceedingly rare, but reports have identified cases among infants with viral infections, primary and secondary immunodeficiencies, as well as other genetic syndromes [4, 5]. Presenting symptoms vary but can include fever, respiratory distress, cutaneous manifestations (such as annular skin lesions involving the eyelids), labial swelling, and extracutaneous manifestations, chiefly gastrointestinal, such as hematemesis, hematochezia, perianal lesions, and hepatosplenomegaly. Generally, treatment is dependent on the underlying disease, and outcomes vary widely from complete recovery among those with viral etiologies, to childhood death in cases associated with underlying congenital disorders.

### Mevalonate kinase-associated diseases

Mevalonate kinase-associated diseases (MKAD) are rare autoinflammatory disorders caused by pathogenic variants in the mevalonate kinase (*MVK)* gene. This gene encodes the mevalonate kinase enzyme, which is responsible for converting mevalonate into a precursor for synthesis of cholesterol and nonsterol isoprenoids [6]. Variable expressivity is a marked feature of MKAD with residual enzyme activity correlating with disease severity. The less severe form, Hyper IgD Syndrome (HIDS), has been associated with up to 28% enzyme function, whereas the more severe form, mevalonic aciduria (MA), has been associated with < 0.5% enzyme function [7]. Infants with HIDS typically present within 1 year of life with recurrent autoinflammatory attacks characterized by fever, abdominal pain, adenopathy, rash, and arthralgias that occur spontaneously every 4-6 weeks or when provoked by physiologic stress (illness or vaccination), and these episodes usually resolve in 3-7 days without treatment [6]. Patients with the MA form typically present in the first few months of life with facial dysmorphisms, ataxia, seizures, myopathies, developmental delay, and failure to thrive along with autoinflammatory attacks [8, 9].

The spectrum of MKAD continues to expand, and more recently MKAD has been identified among cases of very early onset inflammatory bowel disease (VEO-IBD). Briefly, VEO-IBD is defined as IBD occurring before 6 years of age. Although a majority (> 70–80%) of VEO-IBD patients do not have a specific genetic etiology, monogenetic causes are more common in VEO-IBD compared to IBD and include inborn errors of immunity [10].

There are few studies exploring the intersection of MKAD and VEO-IBD. One study examining 10 cases of VEO-IBD among pediatric patients with MKAD found that patients typically presented with abdominal pain, diarrhea, vomiting, and occasionally acute abdomen and anal fistulae [11].

In this case report, we describe the presentation, multidisciplinary approach and management of an infant with MKAD and VEO-IBD who first presented with a rash consistent with Sweet syndrome.

## Case presentation

The patient, born at 35 weeks gestational age with an otherwise unremarkable perinatal course, began having flecks of blood in the stool at 15 days of life initially attributed to milk protein enterocolitis, which continued despite dietary interventions. At 5 weeks of age, the patient presented to the emergency room with a fever to 100.6 °F, tachycardia, and a new rash prompting hospital admission. Admission physical examination showed erythematous, edematous, annular plaques on the anterior neck and bilateral eyelids, scattered erythematous papules on the right neck and extremities, dactylitis of the fingers and toes, and erythematous patches on the buttocks.

Initial blood cell counts showed a significant leukocytosis, with white blood cells (WBCs) 19.36 K/cmm and a neutrophilic predominance (67%), along with anemia (hemoglobin 6.3 gm/dL), which prompted immediate transfusion. Full iron studies were not completed prior to transfusion, but the ferritin level was normal (260 ng/mL). A complete metabolic panel was within normal limits. Urinalysis was notable for 3 + leukocyte esterase, 1–2 + gross blood, 3–10 red blood cells (RBCs) and 4–10 WBCs. The stool was positive for occult blood, and fecal calprotectin was elevated (2433 mcg/g).

Inflammatory workup showed an elevated c-reactive protein to 189.5 mg/L (normal: < 10 mg/L) and erythrocyte sedimentation rate elevated to 72 mm/hr (normal: 0–20 mm/hr).

Infectious workup was largely unrevealing. Procalcitonin was within normal range, at 0.15 ng/mL. Blood and fecal cultures returned negative. Respiratory viral panel including parvovirus type 1–4, rhinovirus, metapneumovirus, adenovirus, influenza A and B, and respiratory syncytial virus was negative. Epstein-Barr virus (EBV) testing was inconclusive (EBV VCA IgM and EBNA IgG were negative and EBV VCA IgG was positive). Human immunodeficiency virus testing was negative. Herpes simplex 1 and 2 PCR were negative.

An extensive immunologic and rheumatologic workup was pursued. Complement component 3 (C3) was elevated to 256 mg/dL, while complement component 4 (C4) was within normal limits. Immunoglobulin (Ig) levels were elevated for age, specifically with IgG 786 mg/d, IgA 77 mg/dL, and IgM 148 mg/dL. Absolute numbers of T cells, B cells, and natural killer (NK) cells subsets were all within normal limits. Rheumatologic workup was negative for anti-nuclear antibodies (ANA), Sjogren’s syndrome A (SSA), Sjogren’s syndrome B (SSB), and ribonucleoprotein (RNP) antibodies.

After consultation with dermatology, a skin biopsy was performed to provide additional diagnostic clarity. Pathology was notable for a dense, superficial to mid perivascular and interstitial mixed inflammatory infiltrate, as well as papillary dermal edema. Given the presence of papillary dermal edema, the biopsy was thought to be consistent with a diagnosis of Sweet syndrome. The patient was started on steroids (methylprednisolone 5 mg/kg daily for 3 days with taper) with an excellent response, including complete resolution of hematochezia and rash.

The patient was discharged, and steroids were tapered as an outpatient. Unfortunately, the hematochezia returned and the patient developed lower extremity edema and labial swelling, prompting transfer to our institution at 2.5 months of age for further subspecialty involvement and ongoing diagnostic workup.

Hematology was consulted for input regarding the patient’s refractory anemia, and overall diagnosis, given the association between Sweet syndrome and hematologic malignancies in the adult population. Peripheral blood smears were reviewed and showed no evidence of malignancy. Bone marrow biopsy was subsequently deferred. Iron studies were difficult to interpret given history of recent blood transfusion, but haptoglobin and lactate dehydrogenase (LDH) were not consistent with hemolysis. Taken together, the patient’s anemia was thought to be secondary to ongoing microscopic gastrointestinal bleeding.

Gastroenterology was consulted, given the association of Sweet syndrome with VEO-IBD. A diagnosis of milk protein enterocolitis was considered. The team recommended sigmoidoscopy with biopsy of the gastrointestinal mucosa after discontinuation of steroid treatment for further diagnostic clarity.

After discussion with the multidisciplinary team, high-dose steroids were discontinued under close observation. The patient was transitioned to physiologic-dose steroids with subsequent recurrence of hematochezia and small pink papules bilaterally on the inner corners of the upper eyelids.

**Fig. 1:**
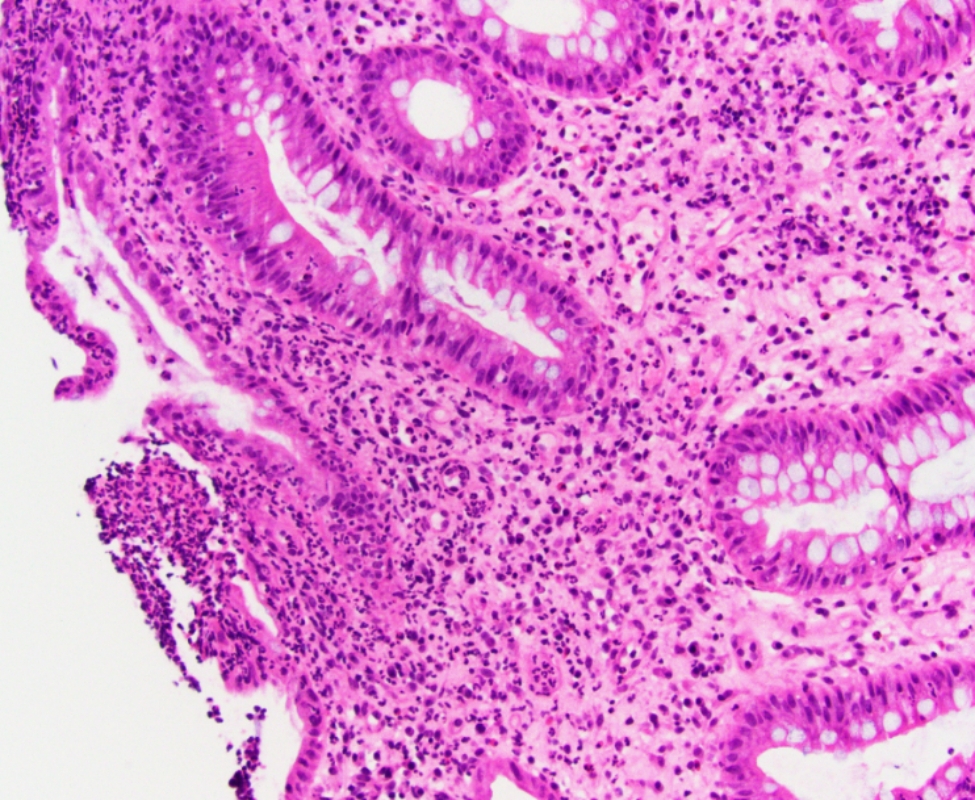
Colonic mucosa biopsy showing intraepithelial acute inflammation and focal erosion (hematoxylin and eosin stain, x20)

Sigmoidoscopy revealed severe inflammation and distinct ulcerations in the descending colon with normal sigmoid and rectal mucosa. Histologic examination of the descending colon biopsy showed severely active colitis with focal erosion and ulceration (Fig. 1). The sigmoid and rectal mucosa were normal.

**Fig. 2:**
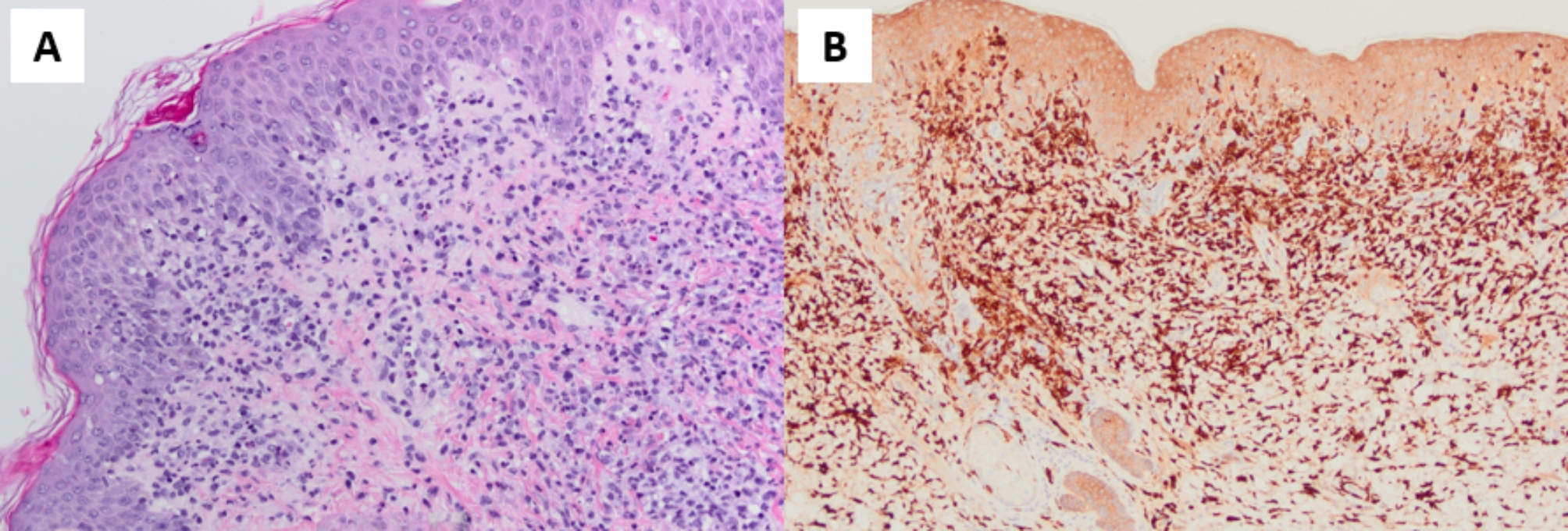
Skin biopsy showing **A**) pityriasiform dermatitis with mild hyperkeratosis, mild spongiosis, focal exocytosis with perivascular and diffuse superficial to mid dermal mixed inflammatory infiltrate with rare eosinophils, and papillary dermal edema (hematoxylin and eosin stain, x20) **B**) CD163 stain highlighting a majority of histiocytic cells in the dermal infiltrate (CD163 stain, x20)

Following the sigmoidoscopy, enteral prednisolone 1 mg/kg daily was prescribed with subsequent resolution of bloody stools. She was discharged home with close outpatient follow-up. Shortly after discharge, pathology skin biopsy slides, which were initially prepared at the outside hospital but were treated with new stains, returned with findings consistent with histiocytoid Sweet syndrome. These findings included epidermal changes with focal scant parakeratosis, focal basal layer vacuolization, a dense mixed dermal infiltrate, and papillary dermal edema with a majority of the cells being CD163 positive (Fig. 2).

Subsequently, targeted sequencing of 594 genes associated with in inborn errors of immunity revealed compound heterozygous, pathogenic variants in *MVK*, c.803T > C, p.268IleThr and c.1129G > A, p.Val377Ile, confirming a diagnosis of mevalonate kinase deficiency (MKD). The patient was started on canakinumab, a human anti-interleukin-1ß (IL-1ß) monoclonal antibody, at standard dosing for treatment.

## Discussion

Here, we present a patient with a rare diagnosis of biopsy-confirmed histiocytoid Sweet syndrome, in the setting of MKAD with VEO-IBD. To our knowledge, this clinical presentation has not been described in the literature. Prior case studies have characterized an association between VEO-IBD and MKAD, and prior case reports have described “Sweet-like” histopathologic and clinical features in MKD [12]. However, there is no literature combining the three distinct elements of this case: histiocytoid Sweet syndrome, VEO-IBD and MKAD.

Genetic testing from the patient identified two compound heterozygous, pathogenic variants in *MVK*, c.803T > C, p.268IleThr and c.1129G > A, p.Val377Ile, on different alleles. The latter variant has been associated with mild clinical symptoms suggesting it is a hypomorphic variant (only partial loss of gene function). This may explain why the patient has some features associated with autosomal recessive associated MVK disease such as VEO-IBD but not cyclical features. Variable expressivity is a well-known feature of MVK-associated disease and therefore this aspect of her presentation is not unexpected. The p.Val377Ile variant is particularly well-documented as it carried by 0.2% of Europeans and has been reported in ~ 90% of HIDS cases [13–15]. This variant is well-established to have incomplete penetrance, which likely contributes to clinical variability. The p.268IleThr variant is also well-established as being pathogenic [7, 16, 17]. These results confirmed the diagnosis of MVK-associated disease.

Once the diagnosis of MKAD was established, the patient was prescribed canakinumab, a human anti-interleukin-1ß (IL-1ß) monoclonal antibody. Until recently, therapy for MKAD consisted of nonsteroidal anti-inflammatory drugs and/or steroids. Evidence of elevated levels of IL-1ß in MKAD as well as in other autoinflammatory fever syndromes led investigators to study the possible therapeutic role of interleukin-1 inhibitors in these diseases. Building on smaller studies, a randomized control phase III trial (CLUSTER trial (NCT02059291) demonstrated that canakinumab was effective and safe in preventing flares in patients with MKAD [18, 19]. The patient’s initial dose was 20 mg subcutaneously every 4 weeks (3.3 mg/kg). Clinical response was monitored using patient symptoms (rash, bloody stool, and monitoring of her weight) and laboratory findings (namely inflammatory markers, including ESR and CRP). The patient’s canakinumab dose was increased to 25 mg subcutaneously (3.7 mg/kg) every 4 weeks to adjust for weight gain and findings of persistent inflammation on laboratory studies. She has been able to wean completely off of glucocorticoids without any major flares of her disease or subsequent hospitalizations. She has had one episode with elevated inflammatory markers, increased stool volume, and blood-tinged stool which resolved spontaneously. Infectious studies were negative. Given spontaneous resolution, it remains unclear if the episode was a mild flare that did not require intervention vs. an infectious etiology. Overall, canakinumab appears to be highly effective at controlling her disease activity to date.

From a gastroenterologic perspective, the patient’s initial hematochezia was responsive to glucocorticoids but refractory to maternal dietary elimination. While sigmoidoscopy revealed severely active colitis with focal erosion/ulceration in the descending colon suggesting possible VEO-IBD, the diagnosis was in question until genetic testing confirmed MKAD. The patient’s presentation and scope findings were consistent with the Bader-Meunier et al. case series of 10 pediatric patients with MKAD [11], where patients were described as having severe colitis with ulceration, and gastrointestinal biopsies with unique histologic features. These features included small intestine villous atrophy without increase of intraepithelial lymphocytes, lymphoplasmacytic and neutrophil inflammatory infiltrate with cryptic abscesses, and profound, large ulcerations inconsistently associated with perforation, necrosis, and/or glandular apoptosis [11]. Some of these atypical histologic findings were not noted in this patient’s biopsies. However, at the time of the sigmoidoscopy the patient had previously been on glucocorticoids for a prolonged period.

## Conclusions

The association of histiocytoid Sweet syndrome with MKAD and VEO-IBD is presented here for the first time in an infant. With systemic immunosuppression using canakinumab, there was resolution of all presenting symptoms. The case highlights the importance of a multidisciplinary approach to rare diagnoses, and collaboration during cases with significant diagnostic uncertainty.

### Electronic supplementary material

Below is the link to the electronic supplementary material.

Supplementary Material 1

## Data Availability

Data sharing not applicable. No new data were created or analyzed in this study. Data sharing is not applicable to this article.

## Abbreviations

VEO-IBD: Very early onset inflammatory bowel disease
SS: Sweet syndrome
MKAD: Mevalonate kinase-associated diseases
MKD: Mevalonate kinase deficiency
*MVK*: Mevalonate kinase gene
HIDS: Hyper IgD Syndrome
MA: Mevalonic aciduria
EBV: Epstein-Barr virus
VCA: Viral capsid antigen
EBNA: Epstein-Barr virus nuclear antigen 1
C3: Complement component 3
C4: Complement component 4
ANA: Anti-nuclear antibody
SSA: Sjogren’s syndrome A
SSB: Sjogren’s syndrome B
RNP: ribonucleoprotein
IL-1ß: interleukin-1ß
