# Supplementary material for: A case of neonatal sweet syndrome associated with mevalonate kinase deficiency
Source: Pediatr Rheumatol Online J. 2023 Sep 12;21:101. doi: 10.1186/s12969-023-00887-8 (PMC10496215; doi:10.1186/s12969-023-00887-8)
Supplement: Supplementary file 1 — Supplementary Material 1 [file 12969_2023_887_MOESM1_ESM.docx]

Dr. Charles H Spencer	July 27^th^, 2023
Editors-In-Chief
*Pediatric Rheumatology*

Dear Dr. Spencer,

Thank you for the opportunity to revise our manuscript. Please find below our point-by-point responses to the questions raised by the *Pediatric Rheumatology* reviewer team. We appreciate responding to these comments, as our manuscript has been strengthened by doing so.

**Reviewer #1:**

1. **Authors need to modify the term GI (page 5, line18) in gastrointestinal (GI)**

*Thank you, this change has been made in the manuscript.*

2. **Delete the term GI (page 5, line 20) because referred to gastrointestinal and not Gastrenterology**

*Thank you, this change has been made in the manuscript.*

3. **Worldwide, autoinflammatory diseases are managed by rheumatologists. I would not make a distinction in the discussion (page 6, line 17 e line 31). I would write Rheumatology and I would insert both the description of the genetics of MVK and of the anti-IL1 therapy. Alternatively, the authors could replace the subheading immunology with genetics. In fact, the paragraph concerns the genotype-phenotype description of MVK.**

*Thank you for pointing out this distinction. We have decided to remove all section titles and reformat the discussion.*

4. **In the description of a case report it would be important to describe the case with photos or images. In this case report, the description of the skin and/or joint involvement must be accompanied by photos (page 4, lines 24-27 and page 5, lines 8-10) or by the pictures of the skin biopsy (page 4, line 56-60) or sigmoidoscopy biopsy (page 5, lines 29-31).**

*Thank you for this feedback. We have now included pictures of both the colonic mucosal biopsy (Figure 1) and the skin biopsy (Figure 2).*

**Reviewer #2:**

1. **Would consider shortening the background and moving a bulk of it to the discussion.**

*Thank you for your suggestions. The background was shortened, and relevant content was moved to the discussion.*

2. **“One study examining 10 cases of VEO-IBD among patients with MKAD found that patients typically presented with abdominal pain, diarrhea, vomiting, and occasionally acute abdomen and anal fistulae" --> study in adults or peds?**

*The referenced study was among pediatric patients. The manuscript has been updated to clarify this point.*

3. **In the case report itself I would consider writing that the patient did receive canakinumab and the dose, not just in the discussion.**

*The line “Patient was started on canakinumab, a human anti-interleukin-1ß (IL-1ß) monoclonal antibody, at standard dosing for treatment.” was added as the last line of the case report prior to the discussion.*

4. **How was clinical response monitored? Clinical symptoms or any laboratory findings?**

*Clinical response was monitored using symptoms (rash, bloody stool and monitoring of her weight) and laboratory findings (inflammatory markers, including ESR and CRP). This sentence has been added to the discussion for additional clarity.*

5. **The genetic testing was completed after starting canakinumab?**

*Canakinumab was initiated after the genetic testing returned, confirming a diagnosis of MKAD.*

6. **Line 35/36 - "Pathology skin biopsy", the p in pathology should be lower case**

*This change has been made in the manuscript.*

7. **Would clarify how mevalonate kinase deficiency (MKD) is different than Melavonate kinase-associated diseases MKAD? Gets confusing since the names are similar.**

*Mevalonate kinase-associated diseases (MKAD) is the broad term encompassing all diseases due to mevalonate kinase enzyme deficiency. Hyper IgD Syndrome (HIDS), also known as Mevalonate kinase deficiency (MKD) is due to mevalonate kinase enzyme deficiency therefore is a type of MKAD. As discussed in the manuscript, the clinical spectrum of MKAD is broad, both in terms of severity and types of clinical symptoms with severity correlating with residual mevalonate kinase enzyme activity.*

8. **If genetic testing was done sooner would it change management?**

*Canakinumab was initiated after the genetic testing returned, confirming a diagnosis of MKAD. Therefore, if testing was done earlier, this medication may have been initiated earlier.*

9. **Is there a possibility that this is primarily MKAD given the response to canakinumab? Is it possible that VEO-IBD and sweet syndrome are manifestations of MKAD?**

*MKAD has been previously reported to cause VEO-IBD. To our knowledge, Sweet Syndrome has not been associated with MKAD, although cutaneous manifestations of MKAD can have “Sweet-like” appearance.*

**Reviewer #3:**

1. **It has been previously reported that cutaneous manifestations of MKAD can have a sweet-like appearance.**

   **a.      Mevalonate kinase deficiency: enlarging the clinical and biochemical spectrum. Prietsch V, et al. Pediatrics. 2003. doi: 10.1542/peds.111.2.258.
   b.      Histopathologic features in a case of hyperimmunoglobulinemia D syndrome. Pace S, et al. Indian Dermatol Online J. 2015. doi: 10.4103/2229-5178.171059.

   Please clarify that the dermatopathology findings in this case have been confirmed as Sweet syndrome and are different from the Sweet-like skin manifestations of MKAD. Could you provide histopathology images and findings?**

*MKAD has been previously reported to cause VEO-IBD. To our knowledge, a diagnosis of histiocytoid Sweet Syndrome has not been associated with MKAD, although cutaneous manifestations of MKAD can have a “Sweet-like” appearance. In this case, the diagnostic criteria for histocytoid Sweet Syndrome was met given the constellation of clinical symptoms in combination with the histologic features (perivascular and diffuse superficial to mid-dermal mixed inflammatory infiltrate, the majority of which were CD163 positive, with rare eosinophils and papillary dermal edema). We have added the second citation to our report.*

2. **Both MVK gene variants identified in this case appear to be known pathological variants. However, have you confirmed that they are present on both alleles? If the patient inherited one variant from each parent, it would confirm their presence on both alleles. It is not ruled out that there could be two variants on the same allele.**

*This is an excellent point. Genetic testing indicated that the variants are on different alleles, with each parent having one of the variants. This information has been added to the manuscript.*

3. **The author suggested that "The latter variant has been associated with mild clinical symptoms suggesting it is a hypomorphic variant (only partial loss of gene function)". Measuring mevalonate kinase enzymatic activity could be important to determine if this variant combination actually has a hypomorphic effect. Please provide information on mevalonate kinase enzymatic activity, IgD levels, or urinary mevalonic acid, if applicable.**

*L Cuissete et al reported a mevalonate kinase enzyme activity of 5% compared to healthy controls in culture lymphocytes from a patient with the same variants as our patient,*

*p.268IleThr variant and p.Val377Ile.*

4. **In the abstract, canakinub should be changed to "canakinumab".**

*Thank you, this change has been made in the manuscript.*

5. **In the Discussion section, comments from each specialty team are included, which is redundant due to some duplication of content. Please summarize each topic on complications or treatment. On the other hand, there is a lack of discussion on skin symptoms (sweet's disease) and no sufficient explanation of the relationship between MKAD and skin manifestations.**

*Thank you for this feedback. We have combined our discussion into a single section, to decrease redundancy. We have now included the dermatopathology pictures, please see response to Reviewer 3 Question 1. We have deleted redundant lines from the previously titled “Gastroenterology” section.*

Sincerely,

| Margaret Irwin, MD  **Boston Combined Residency**  *Boston Children’s Hospital*  *Boston Medical Center* | Veeraya Kate Tanawattanacharoen, B.S.  *Harvard Medical School* | Mollie Wasserman, MD  **Pediatric Hospitalist**  *Boston Children’s Hospital*  *Harvard Medical School* |
| --- | --- | --- |
